# Prevalence of heart failure and coronary artery disease in older adults experiencing homelessness: A systematic review and meta-analysis protocol with a consultative exercise

**DOI:** 10.1371/journal.pone.0345868

**Published:** 2026-06-24

**Authors:** Kristina M. Kokorelias, Yu Qing Huang, Maurita T. Harris, Peter M. Hoang

**Affiliations:** 1 Section of Geriatric Medicine, Department of Medicine, Sinai Health and University Health Network, Toronto, Toronto, Ontario, Canada; 2 Department of Occupational Science & Occupational Therapy, Temerty Faculty of Medicine, University of Toronto, Toronto, Ontario, Canada; 3 National Institute on Ageing, Toronto Metropolitan University, Toronto, Ontario, Canada; 4 Rehabilitation Sciences Institute, Temerty Faculty of Medicine, University of Toronto, Toronto, Ontario, Canada; 5 Department of Medicine, Division of Geriatric Medicine, University of Toronto, Toronto, Ontario, Canada; 6 Institute of Health Policy, Management & Evaluation, Dalla Lana School of Public Health, University of Toronto, Toronto, Ontario, Canada; 7 Faculty of Liberal Arts, Wilfrid Laurier University, Wilfrid Laurier University, Brantford, Ontario, Canada; University of Huddersfield - Queensgate Campus: University of Huddersfield, UNITED KINGDOM OF GREAT BRITAIN AND NORTHERN IRELAND

## Abstract

**Background:**

Heart failure (HF) is a major global public health challenge and a leading cause of morbidity and mortality, particularly among older adults. The rising prevalence of HF is driven by aging populations and the increasing burden of chronic conditions such as hypertension, diabetes, and obesity. In Canada alone, over 750,000 individuals live with HF, with over 100,000 new cases diagnosed annually. Despite advancements in pharmacological and device-based treatments, HF remains a primary cause of hospital admissions, with readmission rates exceeding 20% within 30 days. The associated healthcare costs are projected to reach $2.8 billion annually by 2030.

**Objective:**

Older adults experiencing homelessness represent a particularly vulnerable population at risk for HF. Homelessness is associated with increased exposure to cardiovascular risk factors, including inadequate access to preventive healthcare, high rates of comorbid conditions, and environmental stressors. Evidence suggests that individuals experiencing homelessness have a significantly higher incidence and earlier onset of HF compared to the general population, yet this intersection remains underexplored in the literature. This paper outlines the protocol for a systematic review and meta-analysis that aims to address this gap by synthesizing the prevalence, incidence, and mortality of HF among older adults who have experienced homelessness.

**Methods:**

This systematic review will follow the PRISMA and MOOSE guidelines and has been registered on the Open Science Framework. We will include studies examining the prevalence, incidence, or mortality of HF among older adults (aged 50 and above) with a history of homelessness. The decision to lower the age threshold to 50 reflects the accelerated aging process observed in this population, who often experience age-related conditions at a younger chronological age. Eligible study designs include cross-sectional, cohort, and case-control studies. Quantitative data from peer-reviewed publications and grey literature will be included, with no language or date restrictions. A comprehensive search strategy will be applied across major electronic databases, including Medline, Embase, Cochrane Library, and ISI Web of Science. We will also perform grey literature searches through Google Scholar and governmental websites. Backward citation tracking will be conducted to identify additional relevant studies. Study selection will involve independent screening by three reviewers, with disagreements resolved by consensus. Data extraction will include study characteristics, participant demographics, and HF-related outcomes. Risk of bias will be assessed using the Joanna Briggs Institute Checklist for Prevalence Studies and the ROBINS-E tool for non-randomized studies.

**Results:**

The review will estimate pooled prevalence, incidence, and mortality rates of HF in older adults experiencing homelessness. Where possible, we will conduct subgroup analyses based on age, sex, and comorbidities. Meta-regression and sensitivity analyses will be performed to assess the robustness of the findings.

**Conclusion:**

This systematic review and meta-analysis will provide the first comprehensive synthesis of HF epidemiology in older adults experiencing homelessness. By identifying disparities in cardiovascular health outcomes, this study aims to inform public health policies, healthcare delivery, and future research to improve cardiovascular care for this marginalized population.

## Introduction

Heart failure (HF) is a major and growing public health concern among older adults, contributing substantially to morbidity, mortality, and healthcare utilization [1]. Older adults experiencing homelessness may face an even greater burden of HF and other cardiovascular conditions [2,3]. Structural and social determinants of health associated with homelessness, including limited access to preventive healthcare, high rates of comorbid conditions, challenges with medication adherence, and exposure to extreme environmental conditions, may further compound cardiovascular risk in this population [4]. HF is a progressive, life-threatening cardiovascular condition affecting an estimated 64 million people worldwide [5] Characterized by the heart’s inability to pump blood effectively, HF leads to significant morbidity, frequent hospitalizations, and increased mortality [6]. The condition is particularly prevalent among older adults and individuals with multiple chronic conditions, with incidence rates rising due to aging populations and the increasing prevalence of risk factors such as hypertension, diabetes, and obesity [7]. The prevalence of HF in the homeless population is difficult to quantify; however, emerging evidence suggests higher incidence and earlier onset compared to the general population [2,3]. Studies indicate that people experiencing homelessness have a reduced life expectancy of 17 years [8], with cardiovascular disease—including HF—being a leading cause of death.^2^

In Canada alone, approximately 750,000 individuals live with HF, with over 100,000 new cases diagnosed annually [9]. Despite advances in pharmacologic and device-based treatments, HF remains one of the most common reasons for hospital admissions, with readmission rates exceeding 20% within 30 days [10]. By 2030, the annual healthcare costs related to heart failure in Canada are anticipated to increase to $2.8 billion [11].

In addition to biological vulnerabilities, structural and social determinants of health—such as housing instability, lack of social support, food insecurity, and barriers to accessing primary care—exacerbate the course of cardiovascular disease in this population [2,3]. Many individuals experiencing homelessness rely on emergency departments for fragmented care, leading to delayed diagnoses, poor disease management, and frequent hospital readmissions [12,13]. The absence of stable housing also complicates adherence to self-management strategies [14], which in the context of HF typically require regular follow-ups, lifestyle modifications, and medication titrations [15]. These disparities highlight the urgent need for tailored HF interventions that account for the unique challenges faced by individuals experiencing homelessness.

Despite the growing recognition of HF disparities in marginalized populations, existing HF research often overlooks the intersection of homelessness and cardiovascular health, particularly in the context of older adults. Further, older adults account for 20% of all individuals experiencing homelessness in the United States, and this proportion continues to grow [16]. While numerous reviews have synthesized evidence on pharmacological [17–19], device-based [20, 21], and rehabilitative interventions [22, 23] for older adults living with HF, few have examined how these interventions translate to vulnerable and structurally disadvantaged populations. Moreover, the relationship between effect modifiers—such as age, gender, and comorbidities—and the prevalence, incidence, and mortality of HF in the homeless population is not fully understood. This gap in the literature has limited our understanding of the specific prevalence and severity of HF in individuals experiencing homelessness, particularly among older adults, and the impact of structural factors on these conditions. Further, understanding the epidemiology of heart failure in this population can inform further research, public health policy, and equitable healthcare delivery.

This paper outlines the protocol for a forthcoming systematic review and meta-analysis that seeks to fill this gap by providing a comprehensive analysis of the prevalence, incidence, and mortality of heart failure among older adults who have experienced homelessness. Our review will also evaluate the credibility of effect modification analyses, examining how various factors may influence these outcomes. By synthesizing existing data, we aim to highlight the severity of HF and CAD in this vulnerable population, identify gaps in the current literature, and inform future research aimed at improving care for older adults who have experienced homelessness.

## Methodology

The methods for this study are pre-established in accordance with the PRISMA guidelines for systematic reviews and meta-analyses (PRISMA 2020) and the Reporting Guidelines for Meta-analysis of Observational Studies (MOOSE) [24,25] In the development of this protocol, we have incorporated a consultation phase, which, while not typically included in systematic reviews, draws on recommendations by Buus et al. [26]. Through this phase, we aim to enrich the systematic review by including the perspectives of older adults living with heart failure (HF), as well as a patient partner who identifies as homeless and living with HF, ensuring that the findings are deeply informed by lived experiences. This protocol was created following the PRISMA-P checklist (see supplementary appendix 1). We registered this study on Open Science Framework in March 2025 (osf.io/n924t).

## Methods

### Criteria for considering studies

This systematic review and meta-analysis will focus on older adults aged 50 years and older who have experienced homelessness. The decision to lower the age threshold to 50 is based on the accelerated aging process often seen in individuals experiencing homelessness [27]. Therefore, including individuals aged 50 and above provides a more accurate understanding of the health conditions that disproportionately affect this population.

Studies will be eligible if they assess the prevalence, incidence, or mortality of HF among individuals who have experienced homelessness. This review aims to include studies that provide reliable data on the prevalence of these conditions, as homelessness often complicates access to healthcare and early diagnosis, resulting in higher rates of undiagnosed or advanced-stage cardiovascular diseases in this population. Only quantitative will be included, specifically cross-sectional, cohort, and case-control studies, as these designs are most suitable for evaluating the prevalence of HF and CAD. Case reports, editorials, letters, and non-peer-reviewed sources will also be excluded. We will include abstracts if there are extractable outcomes and search for a published study. We will also complete a search on Google Scholar (n = 300) and governmental organizations for grey literature. Studies conducted in any geographical location will be considered to offer a global perspective on the prevalence of cardiovascular disease in this marginalized population. We will not place any language or search date restrictions. We will contact authors for missing data and will exclude studies for which there are no extractable outcomes. As we will not restrict languages of the studies, we will use Google to translate non-English papers and key information will be verified by bilingual team members where possible to ensure accuracy

### Eligibility criteria

Studies will be included if the majority of participants are aged 50 and above. For studies including younger participants, data for the 50 + subgroup will be extracted where available; if not, studies will be included only if at least 60% of participants meet the age criterion.

### Definitions and outcome measures

In this study, homelessness will be defined as the lack of stable, safe, and adequate housing [28]. For the purposes of this review, this includes individuals living on the streets, in shelters, or temporary housing situations. The definition focuses on those who have experienced homelessness at any point in their lives, with a specific emphasis on older adults aged 50 years and above. In the context of this review, older adults are individuals aged 50 years and older. This age threshold is used to reflect the accelerated aging process often observed in individuals experiencing homelessness, who tend to develop age-related comorbidities at a younger age than the general population [29].

The outcomes of this review will include the prevalence, incidence, and mortality of heart failure among older adults who have experienced homelessness. We define these as point or lifetime prevalence, incidence rates, and excess mortality or standardized mortality, respectively. In studies that have a housed comparison group, we will extract risk or odds ratios, incidence rate ratios, and hazard ratios. This will help identify disparities in cardiovascular health between the two populations. Heterogeneous definitions of heart failure are expected, such as the Framingham criteria or International Classification of Disease (ICD) codes [30].

### Information Sources

Relevant studies for this review will be identified by searching electronic databases, including Medline, Embase, the Cochrane Library, and ISI Web of Science. The search strategy will be developed using a combination of medical subject headings (MeSH) and free-text terms specific to our review’s focus on heart failure (HF), and homelessness among older adults. The search strategy will first be created in Medline and then adapted for use in the other databases. Additionally, we will retrieve further studies through manual reference checking of included studies and relevant reviews using backwards citation sorting [31]. The search strategy will be developed and peer-reviewed using the PRESS (Peer Review of Electronic Search Strategies) guidelines [32], and will be executed by a health service information specialist, who has worked with the research team previously and is familiar with conducting systematic reviews in the medical field.

### Study Records

Studies retrieved from the various databases will be compiled, and duplicate records will be removed using reference management software using the Bramer method (EndNote 2024, Clarivate Analytics) [33]. The identified studies will undergo an independent eligibility review by three authors (KMK, PH, YQH) and a research assistant in a two-step process facilitated by Covidence (Veritas Health Innovation) [34]. Initially, studies will be screened based on title and abstract, and full texts will be retrieved for the second stage. At both stages, intercoder agreement will be evaluated using Cohen’s κ, with a minimum κ value of 0.75 indicating high agreement [35]. Any disagreements between reviewers will be resolved through discussion and consultation with a fourth author (MH).The study selection process will be depicted in a PRISMA flow diagram [24].

### Data Items

Data will be extracted by a research assistant, with oversight from the research team, using a standardized data extraction spreadsheet in Covidence (Veritas Health Innovation) [36]. This spreadsheet will first be piloted on 10 randomly selected studies, and modifications will be made based on this initial review. If further clarifications or additional data are required, the authors of the included studies will be contacted by the research assistant up to three times via email. The data extraction process will include the following key elements: authors’ names and affiliations, year and country of publication, study design, study setting and country, study period, sample size, sampling method, response/adherence rate, and demographic characteristics of participants (such as age, sex, gender, race, ethnicity, and heart failure diagnostic criteria).

We will assess the risk of bias using the Joanna Briggs Institute Checklist for Prevalence Studies [37] and Risk of Bias in Non-randomized Studies of Exposure (ROBINS-E) [38]. We will also apply the Instrument to assess the Credibility of Effect Modification Analyses (ICEMAN) [39] and the GRADE approach [40]. The GRADE framework will be employed to evaluate the quality of evidence across studies, considering factors such as risk of bias, inconsistency, indirectness, imprecision, and publication bias.^40^ Specifically, we will assess how these factors may influence the strength of the findings related to HF in older adults who have experienced homelessness.

### Data synthesis

We will conduct a descriptive analysis to report the characteristics of the included studies through summary tables and narrative text. As we anticipate variability across studies, pooled prevalence estimates for HF will be computed using random-effects meta-analysis models, as this approach is more suitable for handling heterogeneous data compared to fixed-effect models [41]. For meta-analysis of prevalence, we will use a generalized linear mixed effects model using the logit transformation. For meta-analysis of incidence and mortality rates, we will use generic inverse variance. If studies have zero outcomes (e.g., in a comparator group), we will also use generalized linear mixed models. For meta-analysis of mortality rates, we will synthesize the number of deaths divided by the total person-year follow-up time. If follow-up time is not reported, we will estimate the 95% confidence interval before generic inverse variance meta-analysis [42]. In studies that comparator groups, we will estimate the relative risk (e.g., risk ratio, hazard ratio) using generic inverse variance. Heterogeneity will be assessed using the τ^2^, the I^2^ statistic, and the Cochran Q test p-value (<0.05). We will use the Hartung-Knapp adjustment to calculate the confidence intervals around the pooled effects; if there are few studies we will also provide the Wald-type estimator [43]. We will estimate the prediction interval using the t-distribution [44]. We will assess for publication bias using funnel plots.

To address potential sources of heterogeneity, we will perform meta-regression and subgroup analyses where data allow, considering covariates such as age, sex, race, ethnicity, geographical area, diagnostic criteria for HF, and type of heart failure (e.g., reduced ejection fraction and preserved ejection fraction). Cohort studies may differ methodologically from cross-sectional and case-control studies, including follow-up duration and outcome ascertainment; therefore, we will perform a sensitivity analysis excluding cohort studies to assess the robustness of pooled prevalence estimates. We hypothesize that increasing age is associated with heart failure. We will subgroup at the age of 65 as this is the median age of death in this population [45]. We expect that homeless males will have a higher rate of heart failure and mortality than females. While we believe that there are race and gender differences due to structural inequities, we expect a null finding due to study-level measures of race and gender. We expect that there will be a greater proportion of heart failure outcomes in Western and European countries, where individuals experiencing homelessness may have longer life expectancies than low- and middle-income countries. We hypothesize that heart failure with reduced ejection fraction will be more common in this population due to a higher expected prevalence of coronary artery disease.

We will complete the following sensitivity analyses to test the robustness of our findings: removing studies at high risk of bias, removing studies with a sample size under 200, excluding abstracts, and excluding cohort studies. The quality of evidence across studies will be assessed using the GRADE framework, which will help determine the certainty of the findings [40]. This will allow us to assess how these factors may affect the strength of the evidence for the prevalence of HF among older adults experiencing homelessness. Data synthesis will be performed using R version 4.4.1.

### Consultation Exercise

Buus et al. provides methodological guidance to address inconsistencies in the conduct and reporting of consultation phases, particularly in scoping studies [26]. They recommended a mixed-methods study design when integrating a consultation phase into a scoping study.^26^

This study protocol was developed with substantial input from individuals with lived experience, ensuring that the research is grounded in the realities faced by older adults who have experienced homelessness, as well as those living with heart failure and coronary artery disease. The consultation phase, a core qualitative component of our mixed-methods approach, aims to provide deeper insights and contextual understanding that will complement the quantitative data from our systematic review and meta-analysis. This phase specifically incorporates perspectives from those with lived experience, ensuring that their voices and challenges are directly reflected in the study’s findings.

Lived experience experts, including those consulted for our protocol, administrators from organizations that support older adults experiencing homelessness and healthcare professionals, will be invited to a consultation event at the end of the review. A focus group format will be used (conducted in English, which will be spoken by all lived experience individuals) to facilitate group discussion about the study findings. We will share the preliminary findings of the review and ask participants to discuss issues related to counselling based on their expertise and experiences. The qualitative data collected will be analyzed using a reflexive thematic approach, allowing us to identify key themes and categories relevant to the lived experiences of the participants [46].

We will integrate the qualitative findings from the consultation phase with the quantitative results from the systematic review and meta-analysis. By comparing both datasets, we aim to identify areas where the lived experiences of participants expand upon the quantitative data and/or provide recommendations for the implications of findings.

We will follow the guidance of Canham et al. for conducting community consultation to dissemination findings of our review [47]. We will conduct a Knowledge Café, as a community consultation method, using a specific example of exploring stigma and discrimination faced by persons experiencing homelessness [47].

### Dissemination

This review has not began. Results are expected later in 2025. We plan to publish the results in a high-impact, peer-reviewed journal focused on cardiovascular health, public health, or health equity to ensure the findings reach researchers, clinicians, and policymakers (e.g., Journal of the American College of Cardiology, Circulation: Heart Failure, Canadian Journal of Cardiology, BMC Public Health). To maximize accessibility, we will present at national and international conferences, such as the Canadian Cardiovascular Congress, the American Heart Association Scientific Sessions, and the International Conference on Homelessness. Additionally, we will engage with key knowledge users, including healthcare providers, advocacy groups, and policymakers, through tailored policy briefs, infographics, and plain-language summaries.

## Discussion

This protocol outlines the methodology and methods for a forthcoming systematic review and meta-analysis that aims to address a significant gap in the literature by focusing on the prevalence, incidence, and mortality of HF older adults who have experienced homelessness. Individuals experiencing homelessness face numerous health disparities, and the intersection of homelessness with cardiovascular diseases like HF remains underexplored. This review will provide a comprehensive understanding of the extent to which homelessness exacerbates the burden of these cardiovascular conditions, highlighting the unique challenges faced by this vulnerable population. By including both quantitative and qualitative data, we ensure that the lived experiences of individuals with these conditions are considered, thus enriching the evidence base with real-world insights.

Findings from this review have the potential to inform both research and policy. By quantifying the burden of HF among older adults experiencing homelessness, the review will provide evidence to guide healthcare resource allocation, identify priorities for preventive interventions, and support the design of tailored clinical and community-based programs. In addition, highlighting gaps in the current literature can stimulate further research on the intersection of cardiovascular disease and social determinants of health in marginalized populations. Policymakers and service providers may use these findings to implement strategies that improve access to care, optimize management of chronic conditions, and ultimately reduce health disparities in this high-risk group. By integrating both epidemiological data and insights from lived experience, this review contributes a more holistic understanding of HF in the context of homelessness, offering actionable knowledge to improve health outcomes and inform future guidelines and interventions.

This systematic review and meta-analysis offer several strengths. First, it addresses a critical gap in the literature by focusing on the prevalence of heart failure (HF) among older adults who have experienced homelessness, a population that has been largely overlooked in previous research. The inclusion of a consultation phase, which incorporates the perspectives of individuals with lived experience, ensures that the findings are grounded in real-world challenges and needs. Additionally, the review adheres to rigorous PRISMA guidelines and incorporates the GRADE framework to assess the quality of the evidence, ensuring a robust and transparent evaluation process. The decision to focus on older adults aged 50 and above, given the accelerated aging process among individuals experiencing homelessness, enhances the relevance and accuracy of the findings. Furthermore, by incorporating a global perspective through the inclusion of studies from various geographical locations, this review provides a comprehensive understanding of the scope of HF in this marginalized group, facilitating the development of targeted, evidence-based interventions.

This study protocol was developed with substantial input from individuals with lived experience, ensuring that the research is grounded in the realities faced by older adults who have experienced homelessness and who live with heart failure or coronary artery disease. The consultation phase, a core qualitative component of our mixed-methods approach, aims to provide deeper insights and contextual understanding to complement the quantitative data from the systematic review and meta-analysis. Lived experience experts, administrators from organizations that support older adults experiencing homelessness, and healthcare professionals will be invited to a consultation event at the end of the review. Focus groups will be conducted in English to facilitate discussion of preliminary findings and to gather recommendations based on participants’ expertise and experiences. Qualitative data will be analyzed using a reflexive thematic approach, allowing identification of key themes relevant to participants’ lived experiences. While this consultation provides valuable insight into the experiences of older adults experiencing homelessness, it is important to acknowledge that it is specific to the Canadian context and may not fully capture perspectives from other countries or healthcare systems. Additional limitations include the use of English for all focus groups, which may exclude non-English speaking participants, and potential selection bias in the recruitment of lived experience experts. Despite these limitations, integrating consultation findings with quantitative results will enhance the relevance, interpretability, and applicability of the review findings to real-world settings.

Despite the potential implications of this review, several limitations should be considered when interpreting the findings of this review. The exclusion of qualitative studies and case reports may limit our understanding of the broader context of cardiovascular disease among homeless older adults, as these sources may provide valuable insights into the lived experiences of individuals with HF. The reliance on existing studies also means that the quality and consistency of data reporting across studies may vary, which could affect the precision of the pooled prevalence estimates. Moreover, the observational nature of the studies included in this review could result in bias, such as confounding variables that may not be fully accounted for. Accuracy of data from non-English studies may be limited, as not all languages can be verified by bilingual team members. Finally, the generalizability of the findings may be limited by the heterogeneity of the included studies, which may be addressed through meta-regression and subgroup analyses where possible. This review focuses on homelessness in high-income countries; most included studies are expected to represent individuals experiencing street homelessness, and other forms of homelessness, including temporary or insecure housing, may be underrepresented, limiting generalizability.

## Conclusion

This paper outlines the protocol for a systematic review and meta-analysis that will fill a critical gap in the literature regarding the prevalence and incidence of HF among older adults who have experienced homelessness. By synthesizing quantitative and qualitative evidence, the review aims to provide a comprehensive understanding of the cardiovascular health disparities faced by this population. The findings will contribute to the development of targeted interventions to improve healthcare access and disease management for older homeless adults with cardiovascular disease. However, the limitations inherent in the included studies must be considered when interpreting the results, and future research should aim to address these gaps by including more diverse populations and exploring the complex social determinants of health in this context.

## Supporting information

S1 File PRISMA-P 2015 Checklists. (DOCX)
